# Impact of tebipenem pivoxil on the intestinal microbiota and on establishment of colonization with carbapenem-resistant *Klebsiella pneumoniae* in mice

**DOI:** 10.1128/spectrum.02346-24

**Published:** 2025-03-14

**Authors:** Maria F. Mojica, Bryan S. Hausman, Basya S. Pearlmutter, Elizabeth G. Zink, Brigid M. Wilson, Valentina Villamil, Cecilia Saiz, Graciela Mahler, Alejandro A. Vila, Naseer Sangwan, Curtis J. Donskey, Robert A. Bonomo

**Affiliations:** 1 Department of Molecular Biology and Microbiology, Case Western Reserve University School of Medicine, Cleveland, Ohio, USA; 2 Research Service, Louis Stokes Cleveland VA Medical Center, Cleveland, Ohio, USA; 3 CWRU-Cleveland VAMC Center for Antimicrobial Resistance and Epidemiology (Case VA CARES), Cleveland, Ohio, USA; 4 Ohio State College of Medicine, The Ohio State University, Columbus, Ohio, USA; 5 Geriatric Research, Education and Clinical Center, Louis Stokes Cleveland VA Medical Center, Cleveland, Ohio, USA; 6 Laboratorio de Química Farmacéutica, Departamento de Química Orgánica, Facultad de Química, Universidad de la República (UdelaR), Montevideo, Uruguay; 7 Instituto de Biología Molecular y Celular de Rosario (IBR, CONICET-UNR), Rosario, Argentina; 8 Facultad de Ciencias Bioquímicas y Farmacéuticas, Universidad Nacional de Rosario, Rosario, Argentina; 9 Department of Medicine, Case Western Reserve University School of Medicine, Cleveland, Ohio, USA; 10 Lerner Research Institute/Lerner College of Medicine of Case Western Reserve University, Cleveland, Ohio, USA; 11 Department of Pharmacology, Case Western Reserve University School of Medicine, Cleveland, Ohio, USA; 12 Department of Biochemistry, Case Western Reserve University School of Medicine, Cleveland, Ohio, USA; 13 Department of Proteomics and Bioinformatics, Case Western Reserve University School of Medicine, Cleveland, Ohio, USA; Rush University Medical Center, Chicago, Illinois, USA

**Keywords:** tebipenem, metallo-β-lactamase inhibitor, gut microbiome, intestinal colonization, carbapenem-resistant *Enterobacterales*

## Abstract

Tebipenem pivoxil has potent *in vitro* activity against *Enterobacterales* pathogens, but requires combination with β-lactamase inhibitor to achieve activity against carbapenemase producers, including metallo-β-lactamases (MBLs). Herein, we evaluate the potential of tebipenem pivoxil, alone and in combination with the prodrug of the experimental MBL inhibitor CS319 (CS319-piv-SAc), to disrupt the indigenous mice microbiota of the colon and promote colonization by pathogens. The effect of antibiotic treatment (daily for 3 days with subcutaneous saline [control], subcutaneous clindamycin, oral tebipenem pivoxil alone and in combination with CS319-piv-Sac, or oral CS319-piv-Sac) on the intestinal microbiota was assessed by culture for enterococci and facultative Gram-negative bacilli and by 16S rRNA amplicon sequencing. Mice were also challenged with 10,000 colony-forming units (CFU) of multidrug-resistant (MDR) strain *Klebsiella pneumoniae bla*_NDM-1_, 6 h after the second dose. The concentrations of the MDR *K. pneumoniae* in stool were measured on days 1, 3, and 6 after challenge. In comparison to saline controls, clindamycin (*P* = 0.001) and tebipenem pivoxil plus CS319-piv-SAc (*P* = 0.02) treatment resulted in significant changes in the alpha diversity patterns, whereas tebipenem pivoxil and CS319-piv-SAc individual treatments did not (*P* > 0.05). Moreover, clindamycin treatment resulted in substantial overgrowth of MDR *K. pneumoniae* (mean concentration after 6 days of infection, 6.1 vs 2.9 log_10_ CFU/g stool), whereas the other treatments did not (≤3.6 log10 CFU/g). Although tebipenem pivoxil alone or in combination with an MBL inhibitor, CS319, caused alteration of the mice intestinal microbiota, neither treatment promoted overgrowth of carbapenem-resistant *K. pneumoniae*.

**IMPORTANCE:**

In this work, we used a mouse model to determine the impact of tebipenem pivoxil alone and in combination with a prodrug of an experimental metallo-β-lactamase inhibitor, CS319, on the intestinal microbiota and on the establishment of colonization with carbapenem-resistant *Klebsiella pneumoniae*. We found that while treatment with tebipenem pivoxil plus the prodrug of CS319 caused alteration of the intestinal microbiota, it did not promote the overgrowth of carbapenem-resistant *K. pneumoniae*. Although additional studies are needed to examine the impact of tebipenem pivoxil treatment on other multidrug-resistant Gram-negative bacilli, *Clostridioides difficile*, and *Candida* spp., our study is a step forward in the understanding of the potential effect of this oral carbapenem on the indigenous microbiota of the colon and on the promotion of colonization by pathogens.

## INTRODUCTION

A balanced gut microbial community with high richness and diversity plays an important role in maintaining human health. Changes in the microbial population dynamics, represented by loss of diversity and certain cornerstone species, could lead to chronic diseases and opportunistic infections (1). Antimicrobial therapy is considered a major contributor to gut microbiota disruption and has the potential to cause unintended adverse effects by promoting antibiotic-resistant bacteria (1–4). Antibiotics that are excreted in substantial amounts into the intestinal tract are particularly likely to promote the emergence and overgrowth of resistant organisms and *Clostridioides difficile* (2). Therefore, as new antibiotics are developed there is a need to understand their potential to disrupt the indigenous microbiota of the colon and promote colonization by pathogens.

Tebipenem pivoxil, the first oral carbapenem, was developed in Japan and has been marketed there since 2009 for the treatment of pediatric pneumonia, sinusitis, and otitis media (5). Tebipenem is the active form with potent *in vitro* activity against both Gram-positive and Gram-negative bacteria. Gram-positive pathogens susceptible to tebipenem include *Staphylococcus aureus* (methicillin-susceptible), *Streptococcus pneumoniae* (penicillin-susceptible), *S. pyogenes*, *S. agalactiae*, *Enterococcus faecalis*, *E. faecium*, *Bacillus* species, and *Corynebacterium* species (6). Among the Gram-negative pathogens, tebipenem is active against *Moraxella catarrhalis*, *Haemophilus influenzae*, *Neisseria gonorrhoeae*, and *Enterobacterales* pathogens, including extended-spectrum β-lactamase (ESBL) and AmpC producers (5–7). However, tebipenem is inactive against *Pseudomonas aeruginosa* and *Acinetobacter* spp. (8). Furthermore, the acquisition of carbapenemases, such as *Klebsiella pneumoniae* carbapenemase (KPC) and metallo-β-lactamases (MBLs), confers resistance to the antibiotic action of tebipenem (9). Therefore, combination with an appropriate β-lactamase inhibitor is required in these cases.

**Fig 1:**
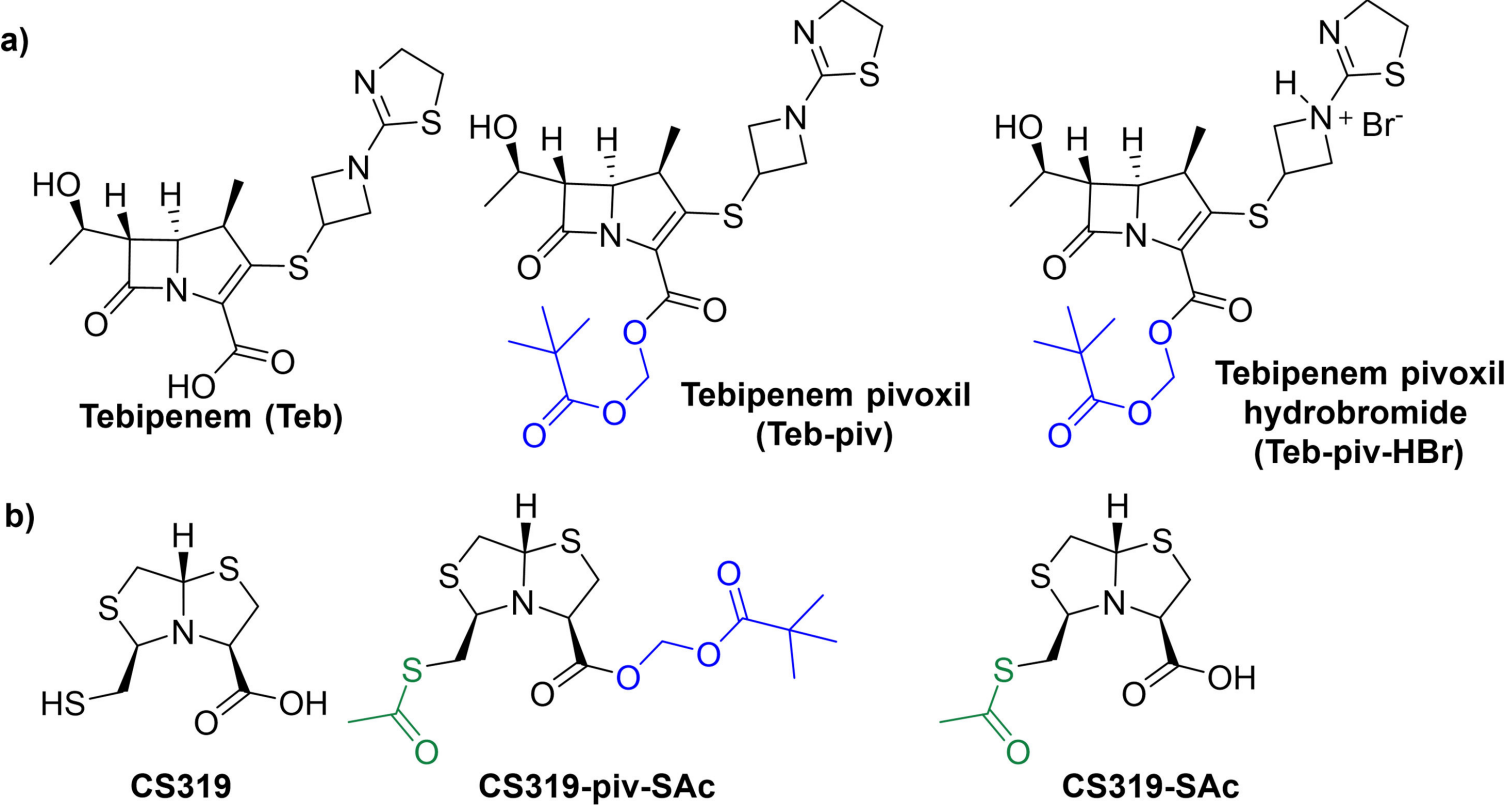
Chemical structures of (**A**) tebipenem and its oral formulations, and (**B**) metallo-β-lactamase inhibitor CS319 and its prodrugs CS319-piv-SAc and CS319-SAc.

In this regard, some of us have been working on the development of cross-class MBL inhibitors for the last decade, such as CS319. Figure 1 shows the chemical structures of tebipenem, tebipenem pivoxil, and CS319 and its prodrugs. CS319 is a bisthiazolidine-based compound bearing both a carboxylic acid and a thiol group, allowing it to work as a competitor inhibitor by forming a reversible complex with different classes of MBLs. CS319 has been shown to restore the activity of imipenem against different Gram-negative bacteria-producing MBLs, including *Escherichia coli*, *K. pneumoniae*, *Acinetobacter baumannii*, and *P. aeruginosa*. Importantly, CS319 has no intrinsic antibiotic activity on its own (10–12). From a pharmacokinetic/pharmacodynamic perspective, the carboxylate group present in CS319 is prone to erratic gastrointestinal absorption, while the thiol group is susceptible to oxidation in biological media, forming disulfide derivatives that lack inhibitory activity against MBL. Therefore, employing a prodrug approach could be beneficial to enhance its physicochemical, biopharmaceutical, and pharmacokinetic properties. Like tebipenem, a pivoxil moiety can be added to the carboxylic acid of CS319 to enhance gastrointestinal absorption. Moreover, strategies to protect the thiol were developed to prevent its oxidation in biological media.

A new formulation of tebipenem pivoxil, tebipenem pivoxil hydrobromide (Fig. 1) has been recently developed and found to be well-tolerated in healthy adults (6, 13, 14). In the recently completed phase 3 clinical trial ADAPT-PO, tebipenem pivoxil hydrobromide was non-inferior to intravenous ertapenem for treating patients with complicated urinary tract infections and acute pyelonephritis. Additionally, the efficacy was similar in both groups regarding fluoroquinolone-resistant, ESBL-producing, and trimethoprim-sulfamethoxazole-resistant strains (13). Therefore, tebipenem pivoxil hydrobromide may address an unmet need for an orally bioavailable carbapenem option to treat serious infections caused by MDR *Enterobacteriaceae*, potentially decreasing the need for intravenous antibiotic therapy in the hospital or outpatient setting. However, there is some evidence that it might have adverse effects on the indigenous intestinal microbiota that could reduce colonization resistance against pathogenic microorganisms (6, 14, 15). In healthy male adults and rats receiving a single oral dose of radiolabeled tebipenem pivoxil hydrobromide, 45–62% of radioactivity was excreted in feces (16, 17). In healthy human volunteers receiving 10 days of tebipenem pivoxil hydrobromide, alteration of the gut microbiome was like alterations caused by amoxicillin-clavulanate, with reductions in *Enterobacterales*, *Enterococcus* spp., *Bifidobacterium* spp., and *Bacteroides* spp (18).

Here, we used a mouse model to examine the impact of tebipenem pivoxil alone and in combination with the CS319 prodrugs (CS319-piv-SAc) on the intestinal microbiota and on the establishment of colonization with carbapenem-resistant *K. pneumoniae*. We found that although tebipenem pivoxil plus inhibitor CS319-piv-SAc treatment causes alteration of the intestinal microbiota, it did not promote the overgrowth of carbapenem-resistant *K. pneumoniae*.

## MATERIALS AND METHODS

### Synthetic procedures

Compounds CS319 and CS319-SAc were prepared according to previous reports (10–12, 19). The synthesis and stability assays of the prodrug form of CS319, CS319-piv-SAc is described in detail in the Supplementary Material.

### The pathogen studied

*K. pneumoniae* Ch1.41 is an MDR strain belonging to the sequence type (ST) 391, which was isolated from an intraabdominal infection. Characterization of the resistance mechanisms in *K. pneumoniae* Ch1.41 by whole-genome sequencing revealed the presence of multiple genes encoding β-lactamases including *bla*_NDM-1_, *bla*_CTX-M-15_, *bla*_DHA_, *bla*_OXA-1_, *bla*_TEM-1B_, and *bla*_SHV-56_; genes encoding aminoglycoside modifying enzymes *aac (3)-IIa*, *aph (6)-Id*, *aph(3″)-Ib*, *aac(6′)-Ib-cr*, and *aac (3)-IId* in addition to *armA*; *fosA6*, *sul1*, *sul2*, *dfrA14*, *qnrB1*, *OqxAB*, and *CatB3* (20). The minimum inhibitory concentrations (MICs) of ceftazidime and imipenem-cilastatin are >128 and >8 mcg/mL, respectively.

### Quantification of stool organisms

Fresh stool specimens were processed as described previously (21–23). Approximately 100 mg of stool was diluted 1:10 in phosphate-buffered saline (PBS), homogenized with a pestle, and serially diluted with 1:10 dilutions in PBS to produce a final dilution of one-billionth of the original concentration. To quantify total Gram-negative bacilli and enterococci, 10 µL samples of the diluted specimens was plated on MacConkey agar (Becton Dickinson, Cockeysville, MD, USA) and Enterococcosel agar (Becton Dickinson), respectively. To quantify *K. pneumoniae* Ch1.41, samples were plated on MacConkey agar containing ceftazidime 6 µg/mL. The plates were incubated for 48 h, and the number of colony-forming units (CFU) of each organism per gram of sample was calculated. All specimens were plated in triplicate.

### Preparation of antibiotics

Clindamycin for intravenous injection (Pharmacia & Upjohn Company, New York, NY, USA) was diluted in PBS. Tebipenem pivoxil (Spero Pharmaceuticals, Cambridge, MA, USA) was initially dissolved in DMSO and the working solution in PBS. CS319-piv-SAc was dissolved in DMSO and the stock solution was mixed in canola oil because it came out of the solution when diluted in PBS.

### Effect of antibiotic treatment on the intestinal microbiota by culture and 16S rRNA amplicon sequencing

For three mice per group, stool specimens (~120 mg total) were collected ~4 h after the second daily dose for analysis of the stool microbiota. An aliquot (~20 mg) of the specimen was plated on selective media as described previously for quantification of enterococci and gram-negative bacilli.

DNA extraction and 16S sRNA amplicon sequencing were performed as described previously (24). Briefly, DNA was isolated from ~100 mg of stool using the QIAamp DNA Microbiome kit (Qiagen). The isolated microbial gDNA was checked for signs of degradation and quantified using the Bio-analyzer (Agilent) to ensure accurate sample input for the initial PCR step. A nested PCR method was used for the amplification of the V4 region of the 16S rRNA gene and the addition of Illumina Nextera Unique Dual indexes. Afterward, each library underwent standard quality control procedures checking for sample concentration and sample quality. Each library was pooled together ensuring equal sample distribution among sequencing reads. Amplicon sequencing was performed on an Illumina MiSeq with a 2 × 150 read length.

### Effect of the antibiotics on establishment of colonization by *K. pneumoniae* Ch1.41

The Animal Care Committee of the Cleveland Veterans Affairs Medical Center approved the experimental protocol. Female CF-1 mice (eight per group) weighing ~30 g (Harlan Sprague-Dawley, Indianapolis, IN, USA) were housed in individual cages. The mice (eight per group) were treated once daily with antibiotics for 3 days. The treatment groups included control (PBS), tebipenem pivoxil 0.9 mg, inhibitor CS319-piv-SAc 2.7 mg, tebipenem pivoxil plus inhibitor, and clindamycin 1.4 mg (positive control). The doses of the drugs were equivalent to human dosing on a mg/kg basis. Clindamycin was administered subcutaneously in 0.1 mL saline. The other antibiotics were prepared as noted previously and were administered by oral gavage in a total volume of 0.2 mL. Control mice received 0.2 mL of canola oil.

Approximately 6 h after the second antibiotic dose, the mice were challenged with 4 log_10_ CFU of *K. pneumoniae* Ch1.41 by oral gavage in 0.1 mL saline. Stool pellets were collected for measurement of *K. pneumoniae* concentrations at baseline and on days 1, 3, and 6 after inoculation of *K. pneumoniae*.

### Data analysis

One-way analysis of variance (ANOVA) was performed to compare concentrations of Gram-negative bacilli among treatment groups after the second daily treatment dose. Dunnett’s test was used for *post hoc* comparison of the antibiotic treatments against the saline control. A non-parametric Kruskal-Wallis rank-sum test was used to compare concentrations of enterococci after the second daily treatment dose because the assumptions of ANOVA were violated. A two-way repeated measures ANOVA was used to compare the impact of the antibiotic treatments on colonization with *K. pneumoniae* Ch1.41 with treatment group and day as significant effects. Dunnett’s test was used as a *post hoc* test to compare the treatments against the saline control. Data analysis was performed in R Version 4.2.2.

For analysis of the sequencing data, individual FASTQ files without non-biological nucleotides were processed using the Divisive Amplicon Denoising Algorithm (DADA) pipeline (25). The output of the DADA2 pipeline (feature table of amplicon sequence variants [an ASV table]) was processed for alpha and beta diversity analysis using phyloseq (26), and microbiomeSeq (http://www.github.com/umerijaz/microbiomeSeq) packages in R. Alpha diversity estimates were measured within group categories using estimate richness function of the phyloseq package. Canonical correspondence analysis was performed using the Bray-Curtis dissimilarity matrix between groups and visualized by using ggplot2 package (27). Differential abundance analysis was performed using ANOVA in R software (The R Foundation for Statistical Computing, Vienna, Austria). As appropriate, we adjusted for multiple comparisons using the BH FDR method while performing multiple testing on taxa abundance across groups (28). Permutational multivariate analysis of variance (PERMANOVA) was performed on all coordinates obtained during CCA.

## RESULTS

### Effect of antibiotic treatment on indigenous Gram-negative bacilli and enterococci by culture

To establish the disruption caused by different antibiotic treatments on the indigenous microbiota and how it may facilitate subsequent colonization by pathogenic bacteria, we first examined changes in the Gram-negative bacilli and enterococci upon administration of clindamycin, tebipenem pivoxil, and tebipenem pivoxil plus the prodrug form of an experimental MBL inhibitor, CS319 (CS319-piv-SAc) versus saline control. As aforementioned, the thiol group of CS319 is prone to oxidation in biological media, forming disulfide derivatives that lack inhibitory activity against MBL. Consequently, we synthesized CS319-piv-SAc, a prodrug derivative of CS319 with a pivoxil moiety attached to its carboxylate group to promote intestinal absorption, and with its thiol protected by an acetyl group (Fig. 1). In aqueous buffer, the prodrug CS319-piv-SAc undergoes slow hydrolysis at the pivaloxyl ester to form CS319-SAc, to a different extent depending on the pH (Fig. S6; Table S1). On the other hand, CS319-piv-SAc rapidly releases the thioacetylated derivative CS319-SAc in human plasma. This derivative, CS319-SAc, is significantly more stable in aqueous buffer and human plasma than the parent drug, CS319. Specifically, CS319 degrades in human plasma with a *t*_½_ = 21 min, whereas the thioacetyl prodrug CS319-SAc is over 20 times more stable, with *t*_½_ = 289 min (Table 1). Therefore, we used the prodrug CS319-piv-SAc for the *in vivo* assays.

**TABLE 1:** Determination of the half-life (*t*_1/2_) of CS319 and its prodrugs derivatives in human plasma

Compound	CS319	CS319Sac	CS319-piv-SAc
Plasma *t*_½_ (min)	21	289	<10

**Fig 2:**
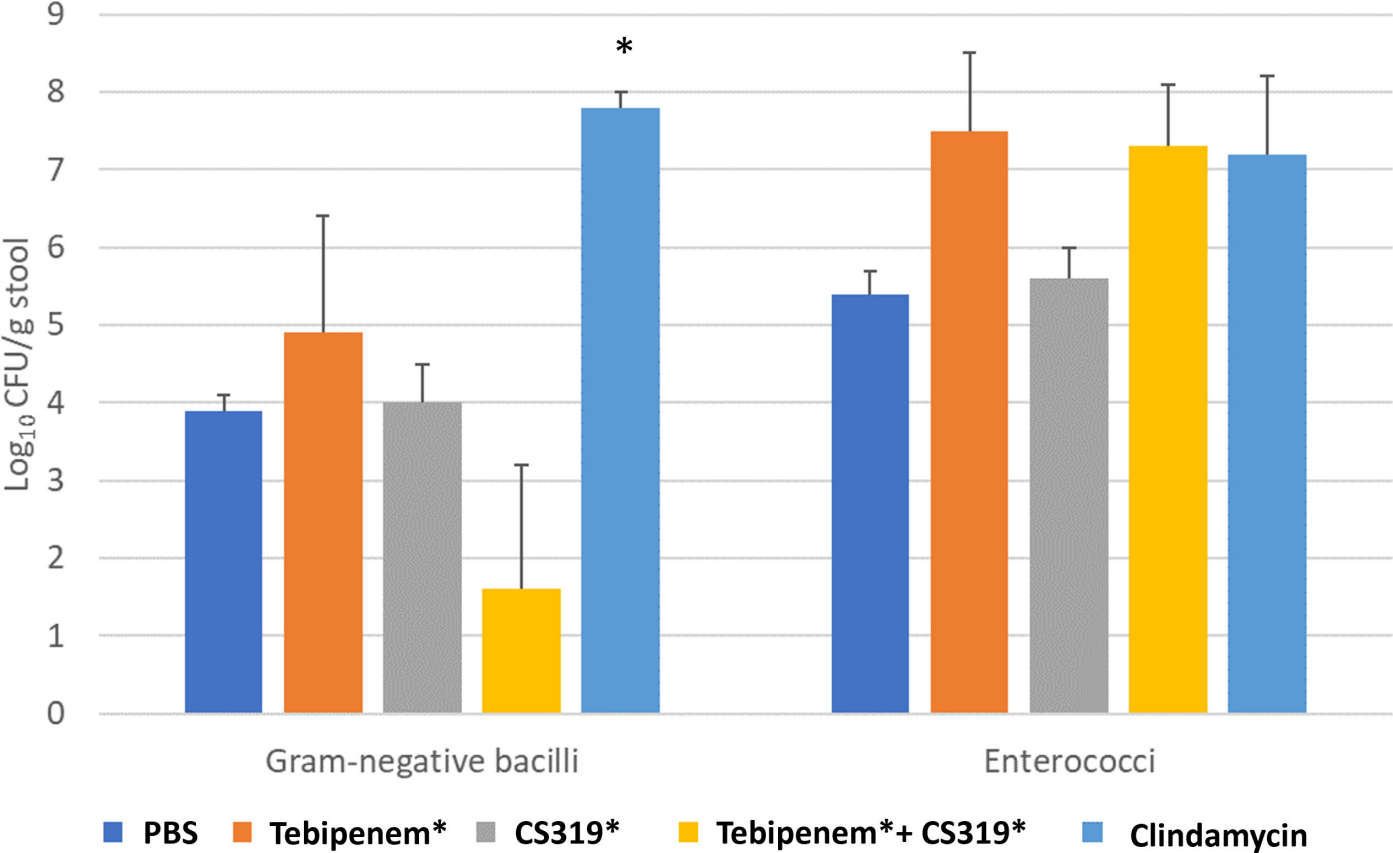
Effect of treatment on the concentrations of facultative gram-negative bacilli and enterococci in stool. Mice (three per group) received daily doses of the treatments for 3 days and stool specimens were collected ~4 h after the second daily dose. Error bars represent standard error.

As shown in Fig. 2, clindamycin treatment resulted in non-significant increases in the concentration of Gram-negative bacilli (*P* = 0.07) and enterococci (*P* > 0.05). In comparison to the saline controls, the concentration of enterococci was ~2 log_10_ higher in tebipenem pivoxil and tebipenem pivoxil plus CS319-piv-SAc-treated mice, but the differences were not statistically significant. Likewise, compared to saline controls, tebipenem pivoxil plus CS319-piv-SAc treated mice had reduced levels of Gram-negative bacilli, but the difference was not statistically significant.

### Sequencing analysis of the stool microbiota

**Fig 3:**
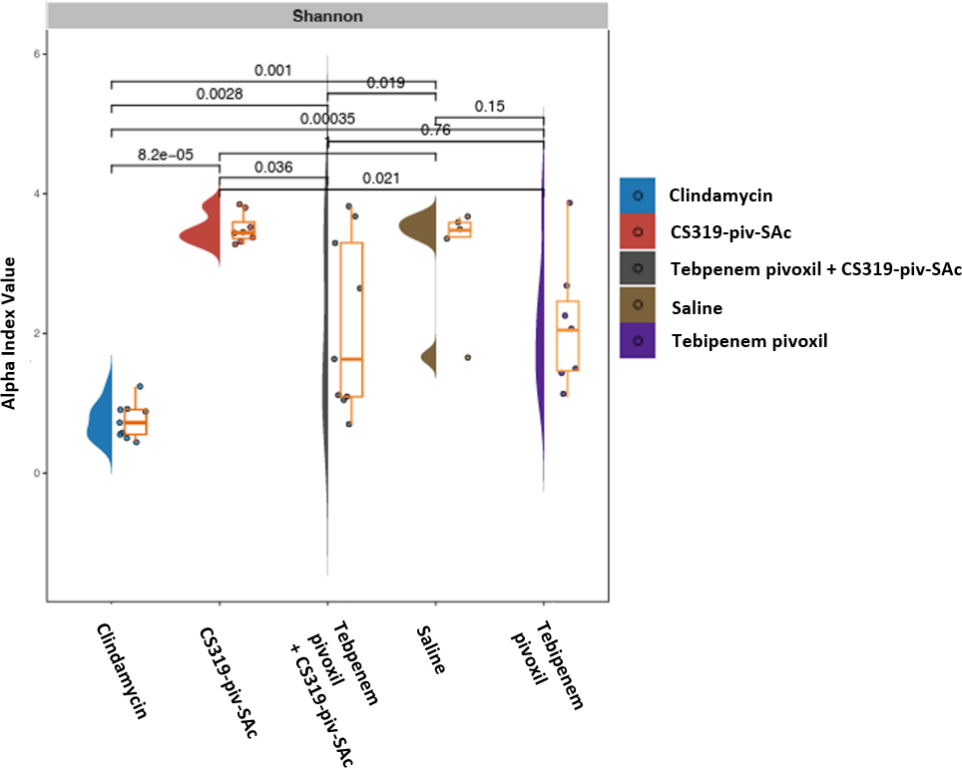
Impact of antibiotic treatment on the total bacterial diversity in the stool of mice as indicated by the Shannon diversity index.

To continue our characterization, we analyzed the impact of treatments on the total bacterial diversity in stool after 2 days of treatment. Alpha (Shannon diversity index; Fig. 3) analysis of 16S rRNA gene amplicon sequencing data revealed differential community shift patterns in clindamycin, tebipenem pivoxil, inhibitor CS319-piv-SAc, tebipenem pivoxil plus inhibitor CS319-piv-SAc, and saline control samples. Clindamycin had the lowest diversity index indicating low species diversity. In comparison to saline controls, clindamycin (Wilcoxon rank-sum test, *P* = 0.001) and tebipenem pivoxil plus inhibitor CS319-piv-SAc (Wilcoxon rank-sum test, *P* = 0.02) treatment resulted in significant changes in the alpha diversity patterns, whereas tebipenem pivoxil and inhibitor CS319-piv-SAc individual treatments did not (*P* > 0.05).

**Fig 4:**
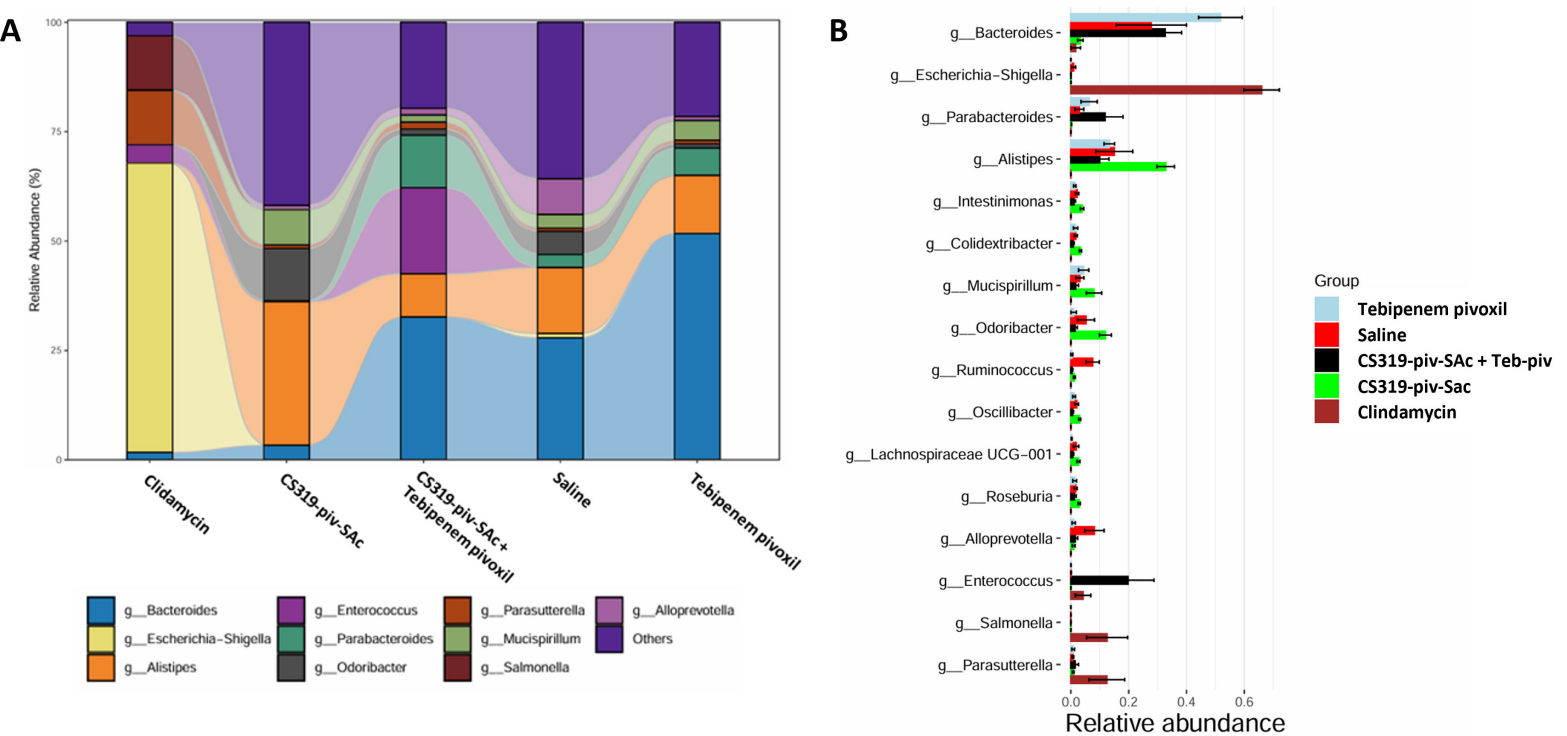
Impact of different antibiotic treatment on the relative abundance of different bacterial genera (**A**). Differentially abundance analysis (*P* < 0.05, ANOVA, Benjamini-Hochberg [BH]) highlighting 19 taxa at genus level whose abundance was differentially altered in the antibiotic treatment groups versus the saline control group (**B**).

Differentially abundance analysis (*P* < 0.05, ANOVA, Benjamini-Hochberg [BH]) highlighted 16 taxa at the genus level whose abundance was differentially altered among the treatment groups (Fig. 4A and B). In comparison to the other groups, clindamycin-treated mice had a marked increase in the relative abundance of several genera in the Proteobacteria phyla, including *Escherichia-Shigella*, *Salmonella*, and *Parasutterella*, whereas there was a decrease in *Alistipes* (phylum Bacteroidota). In comparison to the other groups, tebipenem pivoxil plus inhibitor CS319-piv-SAc-treated mice had an increase in the relative abundance of enterococci.

### Effect of the antibiotics on establishment of colonization by *K. pneumoniae* Ch1.41

**Fig 5:**
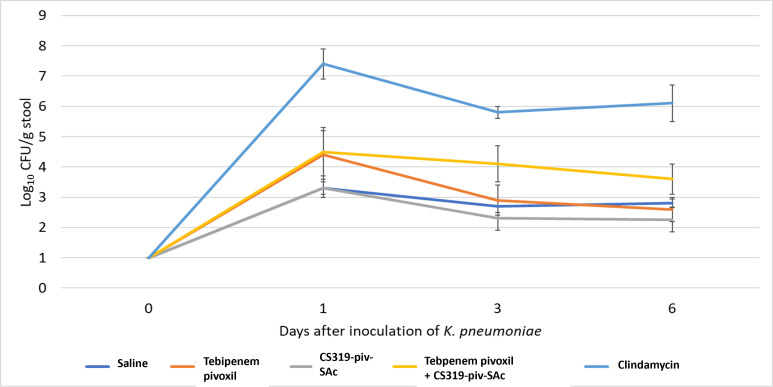
Effect of treatment on establishment of colonization by *Klebsiella pneumoniae*. Mice (eight per group) received 10,000 colony-forming units (CFU) of *K. pneumoniae* 141 by oral gavage on days 2 of 3 of daily antibiotic treatment. The concentration of *K. pneumoniae* in stool was measured at baseline and on days 1, 3, and 6 after gavage. Error bars represent standard error.

To measure the effect of different antibiotic treatments on colonization by pathogenic bacteria, mice were challenged with 4 log_10_ CFU of *K. pneumoniae* Ch1.41 approximately 6 h after the second antibiotic dose. Stool pellets were collected for measurement of *K. pneumoniae* concentrations at baseline and on days 1, 3, and 6 after inoculation of *K. pneumoniae*. None of the mice had detectable levels of ceftazidime-resistant Gram-negative bacilli at baseline. In comparison to saline controls, clindamycin treatment resulted in substantial overgrowth of *K. pneumoniae*, whereas the other treatments did not (Fig. 5). However, ANOVA failed to detect significant differences between the treatment groups and the saline controls (*P* > 0.05).

## DISCUSSION

The gastrointestinal tract and its microbial inhabitants have long been recognized as important components in the host’s health and key indicator of the host’s metabolic status. Furthermore, the gut microbiota plays an integral role in providing resistance against the colonization of pathogenic microorganisms by competition for resources, the production of bacteriocins, and secondary metabolites (29). Thus, disturbance of the usually balanced metabolic state of gut microbiota due to antibiotic therapy, can have significant consequences on the overall health and the disease susceptibility of the host (30). Therefore, understanding the potential impact of new antibiotics on the indigenous intestinal microbiota is important to ensure patient safety.

Previously, a mouse model was used to examine the effect of antibiotic treatment on the establishment and elimination of intestinal colonization with KPC-producing *K. pneumoniae* (31). Results showed that antibiotics such as clindamycin and piperacillin-tazobactam, which inhibit the anaerobic intestinal microbiota *and* have limited activity against the test strain KPC-producing *K. pneumoniae,* promoted the establishment of intestinal colonization with that organism (31). Clindamycin and piperacillin-tazobactam achieve high levels in stool and suppress the anaerobic microbiota of the colon, providing a likely explanation for the high levels of colonization with KPC-producing *K. pneumoniae* they promoted (2, 3).

Clindamycin was used as a positive control for colonization in this study. Compared to the saline control, treatment with clindamycin resulted in significant changes in the alpha diversity patterns of the microbiota and promoted substantial overgrowth of *K. pneumoniae* Ch1.41. Based on differentially abundance analysis, clindamycin treatment resulted in large relative decreases in the relative abundance of members of the class Bacteroidia (*Bacteroides*, *Alistipes*, *Odoribacter*, *Parabacteroides*, and *Alloprevotella*), and of the class Deferribacteres (*Mucispirillum* spp.), with an increase in members of the class Gammaproteobacteria, specifically *Escherichia-Shigella* and *Salmonella*, of the class Betaproteobacteria (*Parasutterella* spp.), and of the class Bacilli (*Enterococcus* spp.). These results are consistent with previous microbiome analyses performed both in mice and humans after clindamycin exposure (2, 3, 32, 33).

The primary aim of this study was to characterize the effect of tebipenem pivoxil alone and in combination with an experimental MBL inhibitor prodrug CS319-piv-SAc on the gut microbiota and establishment of intestinal colonization with an MDR NDM-1 producing *K. pneumoniae* strain. Based on 16S rRNA sequencing analysis, tebipenem pivoxil plus inhibitor CS319-piv-SAc treatment resulted in significant changes in alpha diversity patterns in comparison to saline controls, whereas individual treatment with tebipenem pivoxil or CS319-piv-SAc did not. However, tebipenem pivoxil plus the inhibitor CS319-piv-SAc treatment did not promote significant overgrowth of the carbapenem-resistant *K. pneumoniae* isolate.

Although tebipenem pivoxil alone did not alter alpha diversity in comparison to controls, treatment caused an increase in the relative abundance of members of the class Bacteroidia (specifically *Bacteroides* and *Parabacteroides*), and of the class Deferribacteres (*Mucispirillum* spp.), with an observable decrease in some members of the class Bacteroidia (*Odoribacter* and *Alloprevotella*), and complete eradication of the class Gammaproteobacteria. These results are consistent with previous evidence that tebipenem pivoxil treatment can cause alteration of the intestinal microbiota (18).

The shifts in bacterial populations following treatment with tebipenem pivoxil plus CS319-piv-SAc resemble those observed with tebipenem pivoxil alone, with some differences. While both treatments caused a decrease of the relative abundance of some members of the class Bacteroidia (specifically of the genus *Alloprevotella*), only tebipenem pivoxil plus CS319-piv-SAc caused a significant increase of the class Bacilli (*Enterococcus* spp.), even larger than the increase caused by clindamycin.

Our study has some limitations. We studied the impact of antibiotic treatment on colonization with only one strain of carbapenem-resistant *K. pneumoniae*. However, the level of colonization achieved by the test organism in mice is like levels achieved by other carbapenem-susceptible and carbapenem-resistant *K. pneumoniae* strains (2, 22, 31). Additional studies are needed to examine the impact of tebipenem pivoxil treatment on other multidrug-resistant Gram-negative bacilli, *C. difficile*, and *Candida* spp. The study was conducted using a mouse model with healthy mice dosed once daily with the antibiotics using a dose equivalent to human dosing on a mg/kg basis. The indigenous intestinal microbiota of mice and humans differs, and therefore the findings in mice may not be translatable to findings in humans. Previous studies have demonstrated that administration of antibiotic doses equivalent to human dosing on a mg/kg basis may result in similar drug concentrations in stool (34). However, antibiotic excretion in the intestinal tract of mice and humans may differ. Therefore, additional studies will be required to confirm that the findings are applicable to patients. The challenge with pathogens occurred during antibiotic treatment. Antibiotic-induced disruption of the microbiota may result in a vulnerable period for the establishment of colonization after completion of antibiotic treatment (3). Thus, studies are needed to assess establishment of colonization when pathogen challenge occurs after the completion of treatment. Finally, we did not measure tebipenem pivoxil concentrations in stool. However, although there is evidence that a substantial proportion of tebipenem pivoxil hydrobromide is excreted in stool (14, 16, 17), stool concentrations of tebipenem pivoxil in human volunteers receiving 10 days of treatment were low and often undetectable (16).

## Data Availability

The sequence data for this study can be accessed through the open repository Zenodo and is publicly available at https://zenodo.org/records/14254151.

## SUPPLEMENTAL MATERIAL

Supplemental material spectrum.02346-24-s0001.docx Fig. S1 to S7; Tables S1 and S2.

ASM does not own the copyrights to Supplemental Material that may be linked to, or accessed through, an article. The authors have granted ASM a non-exclusive, world-wide license to publish the Supplemental Material files. Please contact the corresponding author directly for reuse.
