# Supplementary material for: Impact of tebipenem pivoxil on the intestinal microbiota and on establishment of colonization with carbapenem-resistant Klebsiella pneumoniae in mice
Source: Microbiol Spectr. 2025 Mar 14;13(5):e02346-24. doi: 10.1128/spectrum.02346-24 (PMC12054177; doi:10.1128/spectrum.02346-24)
Supplement: Supplemental material — Fig. S1 to S7; Tables S1 and S2. [file spectrum.02346-24-s0001.docx]

**Supplementary Material**

**IMPACT OF TEBIPENEM-PIVOXIL ON THE INTESTINAL MICROBIOTA AND ON ESTABLISHMENT OF COLONIZATION WITH CARBAPENEM-RESISTANT *KLEBSIELLA PNEUMONIAE* IN MICE**

Maria F. Mojica,^1,2,3,4*^ Bryan S. Hausman,^2^ Basya S. Pearlmutter,^2,5^ Elizabeth G. Zink,^6^ Brigid M. Wilson,^6^ Valentina Villamil,^7^ Cecilia Saiz,^7^ Graciela Mahler,^7^ Alejandro A. Vila,^3,8,9^ Curtis J. Donskey,^6,10^ and Robert A. Bonomo^1,3,5,10,11^

**Supplementary Materials and Methods**

**Synthesis**

***General Methods.*** All reactions were carried out in dry, freshly distilled solvents under anhydrous conditions unless otherwise stated. Reactions were monitored by analytical thin layer chromatography (TLC) on 0.25 mm silica gel coated plastic sheets (SIL G/UV 254). Flash chromatography on silica gel 60 (40 µm average particle diameter) was used to purify the crude reaction mixtures. Yields are reported for chromatographic and spectroscopically (^1^H and ^13^C NMR) pure compounds unless otherwise stated. ^1^H and ^13^C nuclear magnetic resonance (NMR) spectra were recorded on a Bruker Avance 400 instrument at 400 and 100 MHz respectively. Chemical shifts (δ) are expressed in parts per million (ppm) downfield from TMS as an internal standard unless otherwise stated. Multiplicities are indicated as s (singlet), d (doublet), t (triplet), q (quartet), m (multiplet), b (broad). Assignments of ^1^H and ^13^C NMR peaks were made based on a combination of COSY, HSQC, and HMBC spectra. Electrospray ESI high-resolution mass spectra (HRMS) were recorded on a MicroTOF-Q spectrometer from Bruker Daltronics. Optical rotation was measured using a Jasco p-2000 polarimeter with a 2.0 mL cell, optical path length of 100 mm and sodium lamp (λ=589 nm) at room temperature. The concentration c is given as g/100 mL. Stability assays were followed by reverse phase high-performance liquid chromatography (HPLC) using a Waters HPLC equipment, with binary pumps (Waters 1525) and photodiode array detector (Waters 2996), with a loop injection of 20 μL (Rheodyne 1727). A reverse phase C18 separation column was used (Kinetex NUCLEOSIL® C18, 150 mm × 4 mm, 5 µm) with detection at λ = 205 nm for all compounds at 37^o^C. The eluent consisted of TFA 0.003 M (mobile phase A) and MeCN (mobile phase B) at a flow rate of 1.2 mL/min. Initial conditions 70/30 (mobile phase A/mobile phase B) changed in 2 min to 55/45, maintained for 3.5 minutes and changed in 0.1 min to 5/95, maintained for 2.4 minutes. Data and chromatograms were collected and analyzed using the Empower System program Waters Corporation, 2002. System linearity was verified in the concentration range: 0.01, 0.041, 0.102, 0.163, 0.203 and 0.244 mg/mL prepared from a stock solution of CS319 1 mM in MeOH. Linearity was established from calibration curve using least squares linear regression analysis and a correlation coefficient (R^2^) value of 0.9991 was found (**Figures** **S1**, **S2** and **S3**). A standard 0.203 mg/mL was injected five times to evaluate precision system, and a % RSD value of 1.0 % was found.

**Synthesis of *tert*-butoxymethyl (*3R,5R,7aS*)-5-((acetylthio)methyl)tetrahydro-5H-thiazolo[4,3-b]thiazole-3-carboxylate (CS319-piv-SAc)**

To a stirred solution of CS319-SAc (150 mg, 1.25 mmol) in EtOAc (4 mL), NaOAc (103 mg, 1.25 mmol) was added. The mixture was stirred at room temperature for 2 h. The solvent was then removed under reduced pressure. The reaction crude was dissolved in DMF (1 mL), and chloromethyl pivalate (242 mg, 1.6 mmol) was added. The reaction mixture was stirred for 24 h. The mixture was poured into a Brine (30 mL), extracted with EtOAc (3 x 50 mL), dried (Na_2_SO_4_) and filtered. The organic layer was evaporated under reduced pressure. The reaction crude was purified by column chromatography on SiO_2_ using n-hexanes/EtOAc (8:2), to afford CS319-piv-SAc (127 mg, 65%) as a white solid; MP = 48-52 ^o^C; ^1^H RMN *δ* 5.82 (d, *J* = 5.4 Hz, 1H), 5.79 (d, *J* = 5.5 Hz, 1H), 5.18 (dd, *J* = 5.2, 2.8 Hz, 1H), 4.31 (dd, *J* = 6.4, 4.5 Hz, 1H), 4.23 (t, *J* = 6.8 Hz, 1H), 3.60 (ddd, *J* = 12.0, 5.2, 0.5 Hz, 1H), 3.26 (m, 3H), 3.09 (m, 2H), 2.34 (s, 3H), 1.22 (s, 9H); ^13^C RMN *δ* 195.5, 177.2, 169.3, 80.0, 74.3, 71.7, 70.3, 38.9, 38.5, 37.9, 34.4, 30.8, 27.0; HRMS calculated for C_15_H_23_NO_5_NaS_3_^+^ [M+Na]^+^ 416.0636, found 416.0636; [α]_D_^20^ = -60.7 (CH_2_Cl_2_, *c* = 2.0). ^1^H and ^13^C NMR spectra are shown in **Figures S4** and **S5**.

Human Plasma stability assays

We assessed the stability of the CS319 and its prodrug derivatives in human plasma. The standard solution was prepared by inactivating human plasma (90 µL) with MeOH (800 µL). Then, phosphate buffer pH 7.4 (100 µL) and a solution of the compound in DMSO (30 mM, 10 µL) were added. The solutions were vortexed, filtered and analyzed by HPLC (A_t=0_).

For the sample preparations, a solution of each compound in DMSO (30 mM, 10 µL) in human plasma (90 µL) and phosphate buffer pH 7.4 (100 µL) was incubated at 37 °C and at suitable time intervals the reaction was stopped by the addition of MeOH (800 µL). The solutions were vortexed and filtered. Degradation time courses were followed by HPLC enabling the quantitation of compounds. The % remain were calculating by area/area percentage, according to:

%remain = (A_t=x_/A_t=0_)*100,

Where A t = 0 corresponds to the peak area of the standard.

Half-lives (t_1/2_) were calculated in Origin using a one-phase decay model with t_1/2_ = ln(2)/b, where b is the slope of a linear plot of natural logarithm (ln) of the remaining compound concentration versus incubation time. Each condition was tested in triplicate.

Buffer stability assays

To measure the stability of the CS319 and its prodrug derivates at different pHs, a standard solution was prepared by adding a solution of each compound in DMSO (30 mM, 10 µL) to a mixture buffer pH 1.2, 4.5, 6.8 or 7.4 (190 µL) and MeOH (800 µL). The solutions were vortexed, filtered and analyzed by HPLC (A_t=0_).

For the sample preparations, a solution of each compound in DMSO (30 mM, 10 µL) was incubated in phosphate buffer pH 1.2, 4.5, 6.8 or 7.4 (190 µL). The solutions were kept at 37 °C and at suitable time intervals the reaction was stopped by the addition of MeOH (800 µL). The solutions were vortexed and filtered. Degradation time courses were followed by HPLC enabling the quantitation of compounds. Half-lives (t_1/2_) were calculated in Origin using a one-phase decay model with t_1/2_ = ln(2)/b, where b is the slope of a linear plot of natural logarithm (ln) of the remaining compound concentration versus incubation time. Each condition was tested in triplicate.

Supplementary Results

Synthesis and Stability Assays of prodrug CS319-piv-SAc

The synthesis of compound CS319-piv-SAc is outlined in Scheme 1.


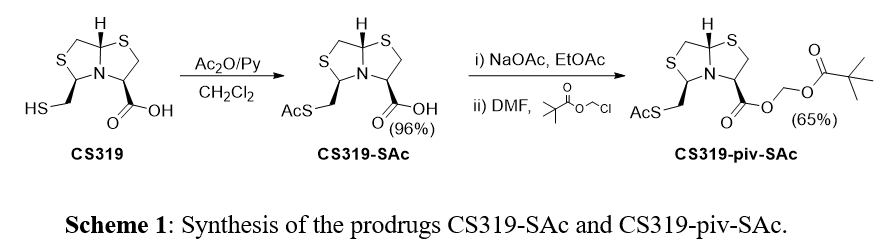


It begins with thiol acetylation using a mixture of Ac2O/Py (1:1), yielding CS319-SAc (96% yield). Next, the sodium salt of CS319-SAc is formed using NaOAc in AcOEt, followed by the addition of chloromethyl pivalate, producing the desired prodrug CS319-piv-Sac in 65% yield.

The in vitro stability was evaluated by incubating the compounds in both buffer (pH 1.2, 4.5, 6.8 and 7.4) and human plasma at 37 °C, and aliquots were taken at different times over 2 h. Plasma and buffer stability samples were analyzed by monitoring the disappearance of the compounds using a validated HPLC technique. Then, the percentage remaining was calculated for CS139, CS319-SAc and CS319-piv-SAc, (Figures S6 and S7, and Tables S1 and S2).

In aqueous buffer, the prodrug CS319-piv-SAc undergoes slow hydrolysis at the pivaloxyl ester to form CS319-SAc, to a different extent depending on the pH. At pH 4.5 and 6.8 the hydrolysis was negligible in a period of 2 h. At pH 1.2 and 7.4, in a period of 2 h CS319-piv-SAC was hydrolyzed by 10 and 13%, respectively (Figure S6 and Table S1).

y = 14.450.496,7335x + 52.105,8715
R² = 0,9999

**Figure S1**: Calibration curve of **CS319-piv-SAc**: Area versus concentration.

y = 20.945.489,754x - 32.295,426
R² = 0,999

**Figure S2**: Calibration curve of **CS319-SAc**: Area versus concentration.

y = 22.382.688,7821x + 43.104,3560
R² = 0,9999

**Figure S3**: Calibration curve of **CS319**: Area versus concentration.


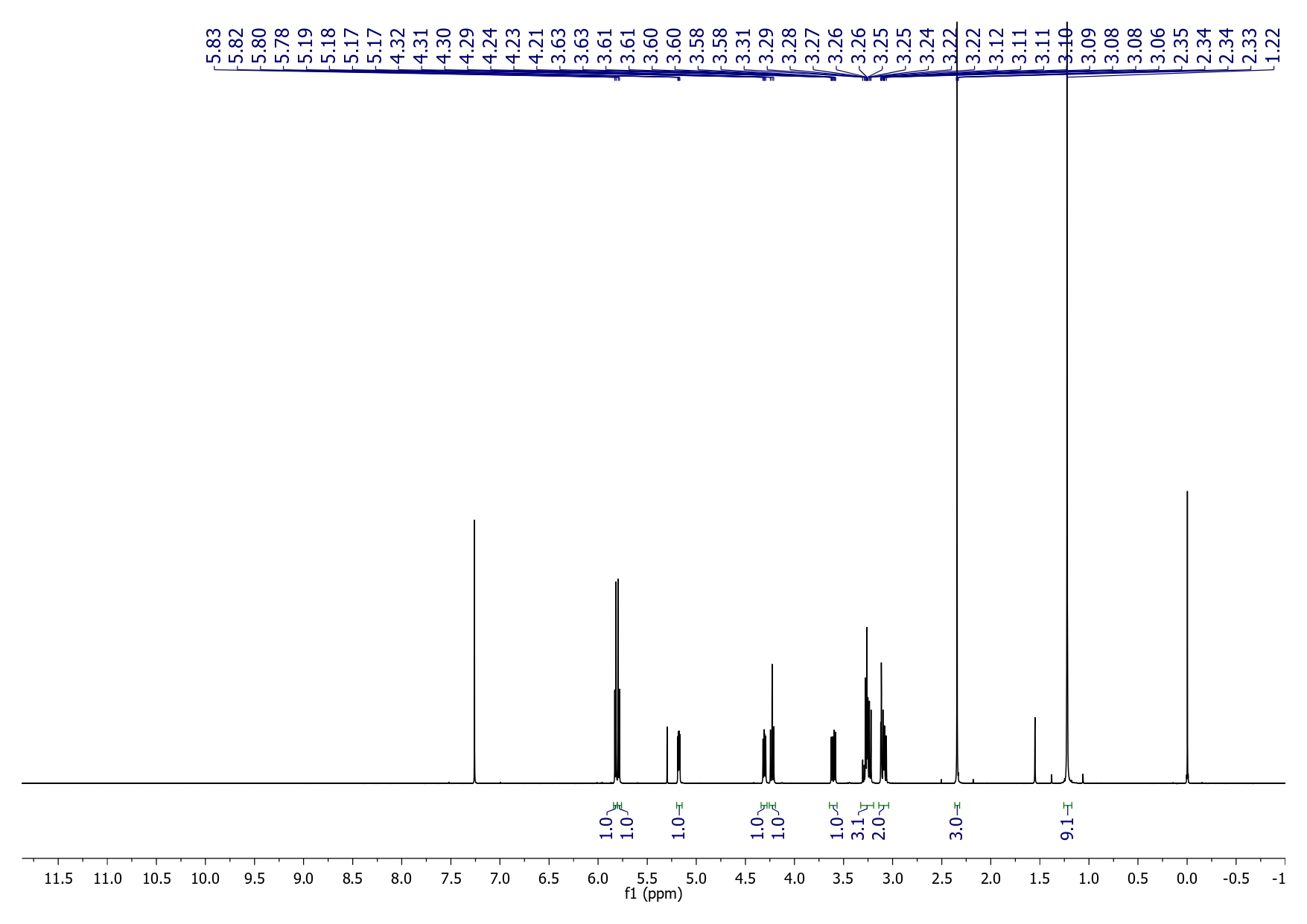


**Figure S4**: ^1^H NMR spectra of compound CS319-piv-SAc.


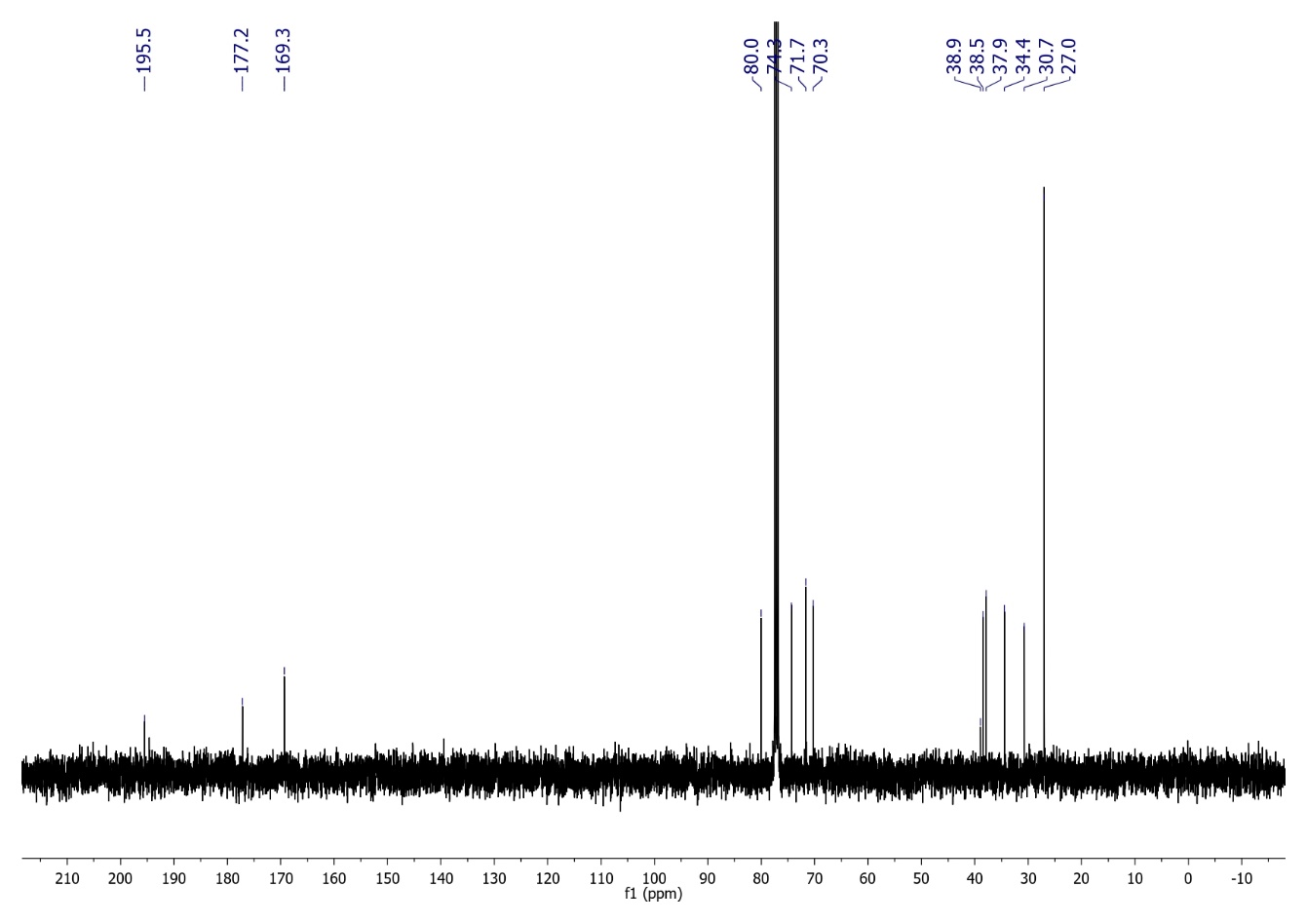


**Figure S5**: ^13^C NMR spectra of compound CS319-piv-SAc.

**Figure S6**: Buffer stability of **CS319-piv-SAc** at pH 1.2 (green), 4.5 (blue), 6.8 (red) and 7.4 (black). Plot % remaining vs time.

**Figure S7**: Human plasma stability of **CS319** (green) and **CS319-SAc** (pink). Plot % remaining vs time

**Table S1**: % Remaining of prodrug **CS319-piv-SAc** at different incubation times in aqueous buffer at pH: 1.2, 4.5, 6.8 and 7.4.

|  |  |  |  |  |
| --- | --- | --- | --- | --- |
|  | **% remaining of CS319-piv-SAc** | | | |
| **Time** (min) | pH 1.2 | pH 4.5 | pH 6.8 | pH 7.4 |
| 15 | 98 | 98 | 99 | 97 |
| 30 | 98 | 99 | 99 | 94 |
| 45 | 96 | 96 | 98 | 93 |
| 60 | 95 | 95 | 98 | 91 |
| 120 | 90 | 96 | 99 | 87 |

**Table S2**: Human plasma stability of **CS319** and its prodrug **CS319-SAc**: % remaining versus at different times.

| **% remaining** | | | | |
| --- | --- | --- | --- | --- |
| **Time** (min) | % **CS319** | t_1/2_ | % **CS319-SAc** | t_1/2_ |
| 15 | 75 | 23 | 98 | 210 |
| 30 | 48 |  | 95 |  |
| 45 | 19 |  | 91 |  |
| 60 | 16 |  | 83 |  |
| 120 | 3 |  | 70 |  |
